# Evaluating different methods used in ethnobotanical and ecological studies to record plant biodiversity

**DOI:** 10.1186/1746-4269-10-48

**Published:** 2014-06-10

**Authors:** Henrique Costa Hermenegildo Silva, Rinaldo Luiz Ferreira Caraciolo, Luiz Carlos Marangon, Marcelo Alves Ramos, Lucilene Lima Santos, Ulysses Paulino Albuquerque

**Affiliations:** 1 Study Group of Ecology and Ethnobiology from the Federal University of Alagoas (UFAL), Campus Arapiraca, Alagoas Zip Code 57309-005, Brazil; 2 Department of Forestry of the Federal Rural University of Pernambuco (UFEPE), Recife, Pernambuco, Brazil; 3 Department of Biological Sciences of the University of Pernambuco (UPE), Campus Mata Norte, Nazaré da Mata, Pernambuco, Brazil; 4 Instituto Federal de Educação, Ciência e Tecnologia de Pernambuco (IFPE), Campus Belo Jardim, Recife, Pernambuco, Brazil; 5 Department of Biology, Laboratory of Applied and Theoretical Ethnobiology (LEA), Federal Rural University of Pernambuco (UFRPE), Recife, Pernambuco, Brazil

**Keywords:** Plant conservation, Semi-arid areas, Ethnobotanical inventory, Vegetation inventory, Parataxonomists, Rapid biodiversity assessment

## Abstract

**Background:**

This study compares the efficiency of identifying the plants in an area of semi-arid Northeast Brazil by methods that a) access the local knowledge used in ethnobotanical studies using semi-structured interviews conducted within the entire community, an inventory interview conducted with two participants using the previously collected vegetation inventory, and a participatory workshop presenting exsiccates and photographs to 32 people and b) inventory the vegetation (phytosociology) in locations with different histories of disturbance using rectangular plots and quadrant points.

**Methods:**

The proportion of species identified using each method was then compared with Cochran’s Q test. We calculated the use value (UV) of each species using semi-structured interviews; this quantitative index was correlated against values of the vegetation’s structural importance obtained from the sample plot method and point-centered quarter method applied in two areas with different historical usage. The analysis sought to correlate the relative importance of plants to the local community (use value - UV) with the ecological importance of the plants in the vegetation structure (importance value - IV; relative density - RD) by using different sampling methods to analyze the two areas.

**Results:**

With regard to the methods used for accessing the local knowledge, a difference was observed among the ethnobotanical methods of surveying species (Q = 13.37, df = 2, p = 0.0013): 44 species were identified in the inventory interview, 38 in the participatory workshop and 33 in the semi-structured interviews with the community. There was either no correlation between the UV, relative density (RD) and importance value (IV) of some species, or this correlation was negative.

**Conclusion:**

It was concluded that the inventory interview was the most efficient method for recording species and their uses, as it allowed more plants to be identified in their original environment. To optimize researchers’ time in future studies, the use of the point-centered quarter method rather than the sample plot method is recommended.

## Background

The consequences of the loss of biodiversity have aroused both interest and controversy [1]. Due to high extinction rates, inventories carried out in the most timely manner possible [1,2] are required to provide rapid and reliable information on the identification and recording of the species present in a particular region.

Questions regarding the accuracy and timeliness of species’ surveys should be considered when choosing the most recommended methods for rapid assessment. Influenced by the Convention on Biological Diversity (CBD) of 1992 [3], such comparisons between methods have occurred since the 1990s [4,5]. Because conservation is, according to Diegues [6], “the management of human use of organisms and ecosystems, to ensure the sustainability of such use,” it would be desirable to incorporate ethnobotanical methods, along with their comparison, into the process of the rapid assessment of plants to clarify which methods are the timeliest and most accurate. Therefore, researchers have compared ethnobotanical methods for inventorying plant species and information regarding vegetation [5,7] that recorded the uses of local vegetation and species, which can lead to more accurate recommendations for their conservation.

To reduce the time spent in data collection in the field, Jinxiu et al. [8] recommended the involvement of parataxonomists, i.e., people who identify biological samples without having had formal training in taxonomy and systematics [9-11]. In addition to aiding with the identification of species samples, parataxonomists have helped to preserve local knowledge.

For local people to recognize biological samples and assist in recording them, the samples can be presented directly in the field by the inventory interview method, which involves a vegetation inventory associated with ethnobotanical data collection [12], similar to the procedure performed by Galeano [13] and Hanazaki et al. [14].

In addition to presenting biological samples in the field, they also can be presented as visual stimuli in the form of ex situ surveys (surveys conducted outside the plant’s original environment), usually in participatory workshops [15]. The tools that have been used as visual stimuli include photographs [12,16], exsiccates [16,17], and fresh material [12,18].

Apart from the involvement of parataxonomists, interviews conducted with a representative group of the population have been an important tool in data collection, allowing the profiling of plant knowledge in the community [19] and assisting in the establishment of conservation strategies using various forms of analysis [20-22]. Abba and Cassini [23] concluded that in addition to providing low-cost and reliable results, interviews effectively contributed to ecological research on three species of armadillos (*Chaetophractus villosus*, *C. vellerosus* and *Dasypus hybridus*) in the Argentinean Pampas and were an important tool in making decisions about soil use and management. Another important methodological aspect to be evaluated is the method of vegetation sampling. In some ethnobotanical studies, it is common to analyze whether the structural composition of the vegetation may be related to the relative importance of the plants for the local community, as supported by the ecological apparency hypothesis [13,24,25]. The use value [25] has been the parameter used to draw conclusions about the cultural importance of certain plants to the human community. However, it is possible that different methods lead to different results. Galeano [13] found a positive correlation between species with the highest use and species that are most important structurally, whereas Tacher et al. [24] found no such relationship. Therefore, due to the importance of the vegetation inventory, there is a need to compare the influence of different methods of sampling vegetation (with the most common being the sample plot and point-centered quarter methods) and their correlation with use value.

Therefore, this study aimed to: a) compare the efficiency of recording species according to methods for accessing local knowledge about native resources used in ethnobotanical studies (semi-structured interviews, inventory interviews, and a participatory workshop with visual stimuli); and b) analyze whether plant recognition is related to the structure of the vegetation, using different methods for inventorying the vegetation (rectangular plots and quadrant points) in areas with different histories of disturbance on the landscape.

## Material and Methods

### Study area

The study was conducted in a village known as *Sítio Carão*, located in the municipality of Altinho, in the state of Pernambuco, Brazil . The village selected for the study is situated 16 km from downtown Altinho, which is located 163.1 km from the state’s capital. At the time of this study, the population of the village was 189 inhabitants (112 were over 18 years of age, consisting of 67 women and 45 men); the local language is Portuguese. The village has an elementary school (to complete their studies, the children travel to downtown Altinho); there is also a Catholic church and a Protestant church [17,26-30]. The central point of the village is located at coordinates 08°35’13.5”S and 36°05’34.6”W.

The economy is sustained by subsistence farming, mainly corn and beans. Livestock farming is restricted to cattle, goats, poultry and a small number of pigs; this type of farming is also responsible for supplementing the food supply and generating family income [17,28-30].

The vegetation consists of *Caatinga*, which is composed of trees that have a maximum height of just over 10 m, with branchy saplings and shrubs that are more abundant. In general, the density of the trees with a trunk diameter more than 3 cm is between 1000 and 3000 per hectare, with basal areas between 10 m^2^ ha^-1^ and 30 m^2^ ha^-1^ and biomass between 20 Mg.ha^-1^ and 80 Mg.ha^-1^ [31]. The climate is dry and the soil is mainly shallow. The area includes the *Sertão* and *Agreste* subzones, the latter occurring in Altinho. As is typical of *Caatinga* vegetation, deciduous, thorny species, and Cactaceae and Bromeliaceae are found in large numbers in the area [32].

The main resources for the village of *Sítio Carão* is the adjacent *Serra do Letreiro* landform, which provides space for agriculture, cattle rearing and the gathering of plants and animals [17,28-30].

### Data collection

For the purpose of this study, three methods were selected: a semi-structured interview with the entire adult population, which requires more time to collect ethnobotanical information; an inventory interview, which requires less time to collect ethnobotanical data but is associated with a previously compiled inventory of vegetation; and a participatory workshop using visual stimuli, which requires the least amount of time to collect ethnobotanical data and which is also associated with a pre-existing inventory of vegetation from which to select the species to be used as visual stimuli.

To assess whether plant recognition is related to ecological apparency, i.e., the structure of the vegetation, or some characteristic related to the population structure of the species, specimens were correlated with the phytosociological parameters obtained from two forms of vegetation sampling carried out in two areas with distinct histories of disturbance, as described below.

**Figure 1:**
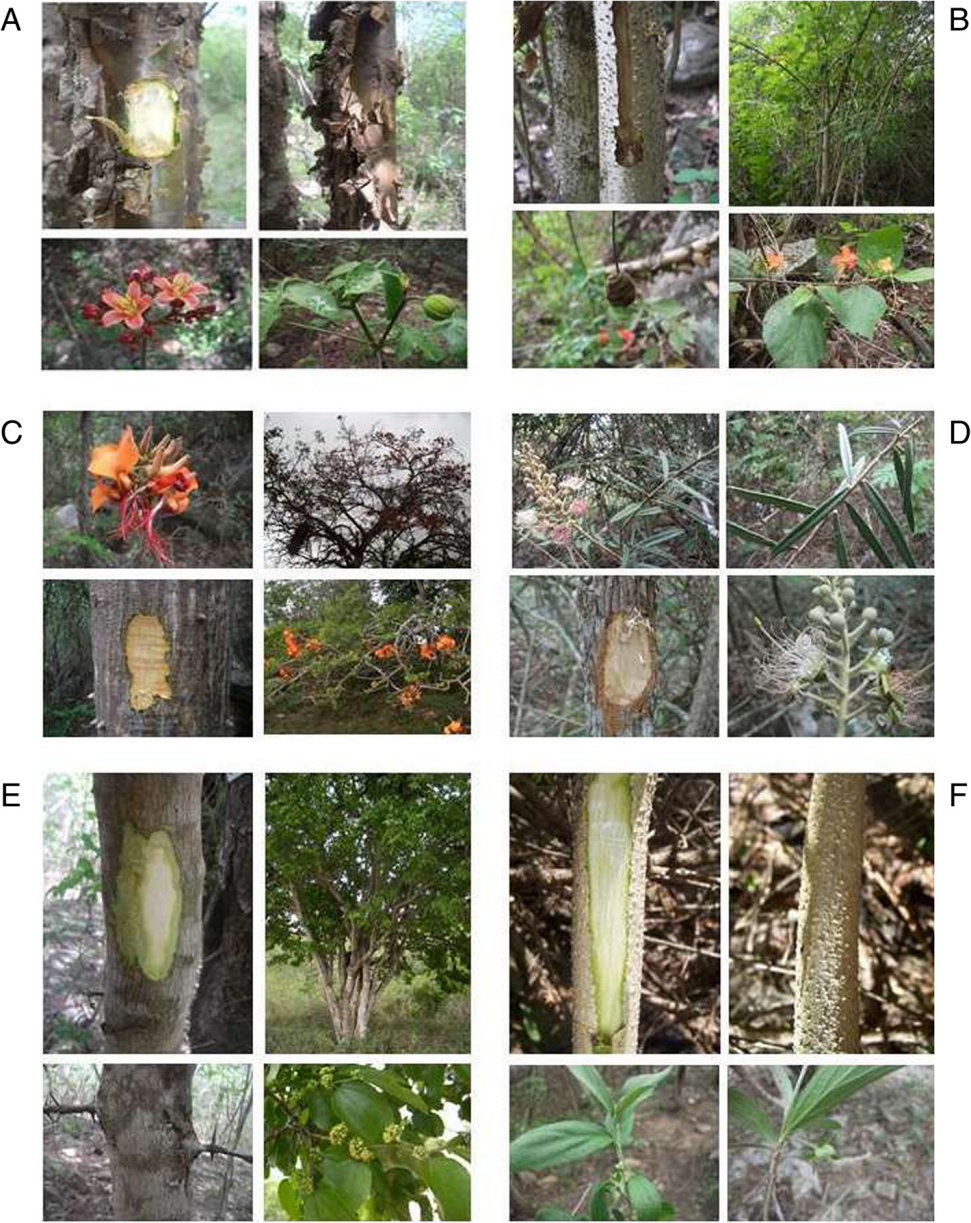
**Photographs of some plant species presented as visual stimuli during the participative workshop in the village of *Sítio Carão*, Altinho municipality, Pernambuco (NE Brazil), in which: a. *Jatropha molíssima *** **(Pohl) Bail. (pinhão bravo); b. *Helicteres macropetala *** **A.St.-Hil. (moleque duro); c. *Erythrina velutina *** **Wild. (mulungu); d. *Capparis jacobinae *** **Moric. ex. Eicler (incó or incol); e. *Ziziphus joazeiro *** **Mart. (juazeiro); f. *Ditaxis malpiguiaceae *** **(Ule) Pax e K. Hoffm. (not recognized).**

To select the plant species presented as visual stimuli (Figure 1), one sample of each of the 62 plant species recorded during a phytosociological inventory was chosen. This field research and inventory were carried out between May 2008 and May 2010 [33]. A criterion for the selected specimens was healthy adult plants, thus the specimens would have the largest possible number of characteristics inherent to the plant structure. A floristic list was prepared using the APG II system, and the names were confirmed from the list of species of the Brazilian flora compiled by the Botanical Garden of Rio de Janeiro [34].

#### ***Semi-structured interview with the community***

Data from semi-structured interviews have been collected since 2006 by the research group of the *Laboratory of Applied and Theoretical Ethnobiology (LEA)* of the Federal Rural University of Pernambuco [17,28-30]. At the time of its implementation, the legal representatives of the Altinho municipality and *Sítio Carão* village were contacted and informed about the objectives of the project, at which point the Family Health Program agents operating in the locality intermediated contact with the informants [17,28-30]. Each person involved was asked to sign a Term of Free and Informed Consent (FICT) (according to resolution 196/96 National Health Service) to authorize the collection, use and publication of data [12]. At this stage in the project, a census was held in which all the people of the village were approached. Thus, all the households in the village were visited, and at least one resident of each household was interviewed. From the total 189 inhabitants, 112 agreed to participate in the survey (men and/or women over 18 years who were heads of households). Regarding the profile of the respondents, over half claimed to be illiterate and to work as farmers; the other half stated that they were involved with various activities in the municipality as a means of earning an income.

Initially, there was a semi-structured interview with each informant to gather information about the popular names of plants in the region (presented in the Table 1), their uses, and collection sites, among other data. The uses of plants cited by informants included the following categories: a) abortive substance - plants used to promote abortion; b) alimentation - this category included all the fruit trees and native plants from which the locals extract resources for human food; c) craftwork - plants that are used in manufacturing handicrafts; d) construction - plants used as elements in building structures that are used for territorial delimitation, e.g., rafters, lines, fences and stakes; e) ecology - plants that serve as food for local wildlife or that have other observed ecological relationships; f) fuel wood - plants used as firewood or charcoal; g) forage - plants used in animal feeding; h) medicinal purposes - plants used as some form of medication for residents; i) technology - refers to the use of plants that undergo transformations from the original raw material but are not designated for use in area delimitation, for example, tool handles and plants used to make furniture; and j) veterinary uses. To ensure that only native plants were examined in the analysis for this study, interviews that exclusively cited plants collected in yards or purchased somewhere out of town were not considered. Of a total of 112 interviews, 88 that mentioned woody plants found on the hills (native vegetation) were used as the basis for the inventory interviews and participatory workshop.

**Table 1:** **Plant species cited in the general interviews, recognized in the participative workshop and inventory interviews with their popular names and respective categories of use (regarded during the survey) in the village *Sitio Carão, *** **Altinho municipality, Pernambuco (NE Brazil)**

**Scientific name**	**Popular name**	**Main uses**
*Amburana cearensis* (Allemao) A.C.Sm.	amburana, umburana açu, cumaru	Alimentation, construction, fuelwood, medicinal purposes, technology, veterinarian uses.
*Anadenanthera colubrina* (Vell.) Brenan	angico	Fuelwood, construction, medicinal purposes, technology, veterinarian uses.
*Bauhinia cheilantha* (Bong.) Steud.	mororó, pata-de-vaca	Alimentation, construction, fuelwood, medicinal purposes, technology.
*Campomonesia* sp.		Alimentation.
*Cedrela odorata* L.	cedro	Construction, medicinal purposes, technology, veterinarian uses.
*Chloroleucon extortum* Barneby e Grimes	jurema branca	Alimentation, construction, forage, fuelwood, medicinal purposes, technology.
*Chorisia speciosa* A.St.-Hil.	barriguda	Alimentation, technology.
*Commiphora leptophloeos* (Mart.) J.B.Gillett	imburana, imburana braba, umburana	Alimentation, construction, medicinal purposes, technology.
*Croton argyroglossus* Baill.	rama branca, sacatinga	Construction, fuelwood, medicinal purposes, technology, veterinarian uses.
*Croton blanchetianus* Baill.	marmeleiro	Construction, fuelwood, medicinal purposes, technology, veterinarian uses.
*Croton heliotropiifolius* Kunth	velame	Medicinal purposes, technology.
*Erythrina velutina* Willd.	mulungu	Alimentation, construction, craftwork, medicinal purposes, technology, veterinarian uses.
*Eugenia pyriformis* Cambess	ubaia	Alimentation, construction, ecology, fuelwood.
*Eugenia* sp.	batinga	Alimentation, fuelwood.
*Guapira laxa* (Netto) Furlan	piranha	Construction, fuelwood, medicinal purposes, technology, veterinarian uses.
*Handroanthus impetiginosus* Mattos	pau d’arco or pau d’arco roxo	Construction, fuelwood, medicinal purposes, technology.
*Jatropha mollissima* (Pohl) Baill.	pinhão bravo, pinhão	Construction, medicinal purposes.
*Libidibia ferrea* (Mart. ex Tul.) L.P.Queiroz	jucá, pau-ferro	Alimentation, construction, fuelwood, medicinal purposes, technology, veterinarian uses.
*Manihot dichotoma* Ule.	maniçoba	Abortive substance, alimentation, fuelwood, medicinal purposes, technology.
*Maytenus rigida* Mart.	bom nome, rompe jibão	Alimentation, construction, fuelwood, medicinal purposes, technology.
*Myracrodruon urundeuva* Allemao	aroeira	Construction, fuelwood, medicinal purposes, technology.
*Myrciaria cauliflora* (Mart.) O.Berg	jabuticaba	Alimentation, medicinal purposes.
*Piptadenia stipulacea* (Benth.) Ducke	amorosa, jurema rasga beiço	Construction, fuelwood.
*Poincianella pyramidalis* (Tul.) L.P.Queiroz	catingueira	Alimentation, construction, fuelwood, medicinal purposes, technology.
*Sapium argutum* (Müll.Arg.) Huber	burra leiteira	Construction, technology.
*Schinopsis brasiliensis* Engl.	baraúna or braúna	Construction, fuelwood, medicinal purposes, technology.
*Senna martiana* (Benth.) H.S.Irwin e Barneby	canafístula, canafista	Craftwork, construction, technology.
*Spondias tuberosa* Arruda	umbu or pé de umbu	Alimentation, fuelwood, medicinal purposes.
*Syagrus cearensis* Noblick.	coqueiro catolé	Alimentation, construction, medicinal purposes, technology.
*Syagrus* sp.	coqueiro miudinho	Medicinal purposes.
*Ziziphus joazeiro* Mart.	juazeiro	Alimentation, construction, fuelwood, forage, medicinal purposes.

#### ***Inventory interview***

An inventory interview [35] was conducted in which two members appointed by the community were brought to the area where we carried out the sampling of vegetation (phytosociological) (see “Vegetation sampling”), and the chosen specimens were presented to the informants in an interview with a runtime of 4 h, in which they were asked about the names of the species and their uses. These informants are locally recognized as having deep knowledge of the local native vegetation. One of them is younger (approximately 20 years old) and has always accompanied and supported our team in field work, and the other one is older (approximately 60 years) and very active, and he walks through local vegetation searching for plant resources.

#### ***Participatory workshop***

The entire community was invited to the workshop on the occasion of each house call; residents were given information on the purpose of the workshop and an invitation with the date and location where it would be held. All the villagers were asked to participate, regardless of whether they were local experts. To conduct the workshop, the participants were divided into three groups to optimize time and facilitate the collection of information. People were drawn at random to assemble the groups proportionally and to form more heterogeneous groups, which were then presented with exsiccates and photographs of the 62 plant species recorded during the phytosociological inventory (see “Vegetation sampling”). The participants were shown exsiccates and photographs of the leaf, bark, inner bark and, when present, flower and fruit using a laptop monitor (Figure 1). The duration of the participatory workshop was 90 min, and the number of participants ranged between 24 and 32, with between 8 and 12 people per group. The variation was due to the arrival of people after the start of the workshop or departure before the end. We asked about the names of the species and their uses during workshop.

As a single botanical species may have different common names within the same community, during its six years of research the team of the Laboratory of Applied and Theoretical Ethnobiology (LEA) have sought to crosscheck the names that local residents have given each plant in the locality. Thus, it was possible to record the correct recognition of the species names by the community using exsiccates and photographs.

#### ***Vegetation sampling***

The criteria used to select the two areas for vegetation sampling were that there was no record of the land being used for agriculture (Area 1) or that the land had been abandoned for nearly 30 years after being used for agricultural planting (Area 2).

There were 20 rectangular plots of 10 m × 20 m installed in each area for a total of 40 plots, constituting 0.8 ha in total. Additionally, 400 point quadrants were installed in each area for a total of 800 quadrant points.

### Analysis of data

To check for significant differences in the proportion of plant species recorded using the investigated methods, Cochran’s Q test was applied using BioStat 5.0 software [36] to analyze the 62 species recorded during the phytosociological inventories in *Serra do Letreiro*. For the analysis, a matrix of present and absent species was created for each method to assess whether there was a significant difference in the proportion of species recorded per method. In this matrix, the present species were those mentioned during the interview and recognized during the participatory workshop or inventory interview. Absent species were those not mentioned in the interview and not recognized in the participatory workshop or inventory interview. For this analysis, three groups were created for each method and the proportion of species in each from among the 62 registered was compared.

Using the results obtained in the general interview, the use value (UV) of the plant species was calculated following an adaptation of the Phillips and Gentry [37] method, as created by Rossato et al. [38] and Silva et al. [39], using the following formula:

$$UV_{\mathrm{is}}=\Sigma U_{\mathrm{is}}/n_{\mathrm{is}}$$

Where *UV*_*is*_ = the use value of the species *s* mentioned by the informant *i*; *ΣU*_*is*_ = the number of uses of species *s* mentioned in each event by the informant *i*; *n*_*is*_ = the number of events in which the informant *i* cited species *s*.

Of the total number of native species recorded (62 were recorded in the inventory of vegetation), 31 species were mentioned during the interview and also recognized during the inventory interview and participatory workshop (Table 1). The use value of this group of species was correlated with the relative density (RD = 100ne/N, where ne = the number of individuals sampled within species e and N = the total number of individuals sampled, regardless of species) and importance value (IV = RD + RF + RBA).

*RFs = 100AFs / ΣAFs*, where *AFs* = the absolute frequency of species *s* (%) and *ΣAFs* = the sum of all absolute frequencies of each species and that RBA = *100BAs*/BA, where BA*s* = the absolute basal area of species *s* and BA = the absolute basal area (m^2^) of all the individual plants, regardless of species, obtained in the inventory of the vegetation plots using the sample plot method and point-centered quarter method in two areas with different disturbance histories.

Although the importance value also considers relative density, other parameters can influence its final value; therefore, the analysis was conducted in this manner to determine whether there were differences in the results obtained from the distinct vegetation sampling methods and those obtained from the different sampling locations. This approach was also used to determine whether there could be a bias in the interpretation of the ecological apparency hypothesis depending on the history of its ecological use in the location sampled and the method used. In addition to these analyses, it should be possible to correlate the identification of species to the structure of the vegetation. This analysis was performed based on the Spearman’s rank correlation coefficient using BioStat 5.0 software [36].

## Results

### Methods used in ethnobotanical studies for accessing local knowledge about native plants

Of the 62 species listed in the participatory workshop, 38 were recognized, nine were mistaken for different species, and 15 were not identified. In the inventory interview, 44 species were identified, 17 were not recognized, and one was confused with another species. Of all the species presented during the participatory workshop and the inventory interview and mentioned during the semi-structured interview, 31 were mentioned in all methods (Table 1), three were recognized only in the participatory workshop, five were recognized only in the inventory interview, and 29 were cited during the semi-structured interview with the community and were not recorded in the phytosociological inventory. The total number of native species recorded by the semi-structured interview was 49. In the inventory interview and participatory workshop, the identity of the plants was previously known by the researchers, and an error was considered to be made when the informants had different names for species than the community had previously submitted.

These differences between the methods are significant. The application of Cochran’s Q test confirmed distinctions between the proportions of species recognized and listed in the methods (Q = 13.37, df = 2, p = 0.0013), with the inventory interview being the most efficient method for recording useful species.

### Relationship between use value and vegetation structure from the application of different methods for inventorying the vegetation in places with different histories of disturbance

The species with the highest use values (UV) were *Chloroleucon extortum* Barneby and Grimes, *Calliandra* sp., *Croton blanchetianus* Baill., *Libidibia ferrea* (Mart. ex Tul.) L.P.Queiroz and *Chorisia speciosa* A.St.-Hil, all of which had a UV above 1 (Table 2). The results obtained from the Spearman’s rank correlation coefficient comparing UV, RD and IV obtained from the sample plot and point-centered quarter methods indicated no correlation between UV, IV and RD for the sample plot method in Area 1 (rs = 0.25, p = 0.22 and rs = -0.18, p = 0.35, respectively) or between RD, UV and IV for the point-centered quarter method in Area 1 (rs = - .12, p = 0.56 and rs = -0.08, p = 0.7, respectively). This result suggests that species with a high UV in Area 1 are not the most abundant.

**Table 2:** Data about the use value (UV) obtained in the general interview with the community and the relative density (RD) and importance value (IV) for the two phytosociological methods studied in the two areas

		**PA1**	**QA1**	**PA2**	**QA2**	**PA1**	**QA1**	**PA2**	**QA2**
	**UV**	**RD** ***s***	**RD** ***s***	**RD** ***s***	**RD** ***s***	**IV**	**IV**	**IV**	**IV**
*Amburana cearensis* (Allemao) A.C.Sm.	0.5	0.1	0.8	-	-	1.04	1.99	-	-
*Anadenanthera colubrina* (Vell.) Brenan	0.75	0.19	0.8	0.05	0.27	0.87	2.54	0.59	2.42
*Bauhinia cheilantha* (Bong.) Steud.	0.82	11.13	9.61	4.3	4.52	19.73	20.93	15.91	13.62
*Libidibia ferrea* (Mart. ex Tul.) L.P.Queiroz	1.15	0.1	3.47	-	0.13	0.44	10.19	-	2.44
*Poincianella pyramidalis* (Tul.) L.P.Queiroz	0.5	4.13	3.47	6.56	7.18	9.74	8.28	23.63	68.24
*Calliandra* sp.	1	1.73	-	-	-	4.29	-	-	-
*Cedrela odorata* L.	1.58	1.63	2.54	-	-	8.1	18.51	-	-
*Chloroleucon extortum* Barneby e Grimes	0.8	2.69	4.14	-	-	7.13	12.15	-	-
*Chorisia speciosa* A.St.-Hil.	2	0.19	0.93	0.05	0.13	0.51	4.58	1.2	0.51
*Commiphora leptophloeos* (Mart.) J.B.Gillett	1.07	3.36	7.21	3.17	5.05	13.13	28.13	21.42	17.26
*Croton argyroglossus* Baill.	0.56	5.66	3.47	3.55	2.53	10.84	7.87	8.53	6.71
*Croton blanchetianus* Baill.	0.65	8.83	7.21	59.6	55.19	17.67	17.14	115.81	112.3
*Croton heliotropiifolius* Kunth	1	0.1	-	0.11	0.27	0.44	-	0.6	0.5
*Erythrina velutina* Willd.	1.25	0.86	0.93	-	0.13	4.03	5.01	-	0.39
*Eugenia* sp.	1	3.36	2.67	4.25	0.66	8.25	7.52	17.47	2.26
*Guapira laxa* (Netto) Furlan	1	2.3	0.93	0.05	-	6.18	2.38	0.55	-
*Jatropha mollissima* (Pohl) Baill.	0.6	2.78	2.94	1.29	2.79	6.95	6.65	7.53	7.68
*Ziziphus joazeiro Mart.*	1	-	-	0.05	-	-	-	0.63	-
*Manihot* sp.	1	2.11	4.14	0.48	0.43	6.09	10.99	3.12	2.21
*Maytenus rigida* Mart.	0.82	0.19	0.53	-	-	0.53	2.34	-	-
*Myracrodruon urundeuva* Allemao	0.93	2.11	3.34	1.72	2.36	7.93	10.28	12.29	7.03
*Myrciaria cauliflora* (Mart.) O.Berg	0.5	1.25	2.4	0.16	1.24	3.92	7.95	0.75	8.54
*Piptadenia stipulacea* (Benth.) Ducke	0.63	1.92	6.14	1.18	0.25	4.27	14.96	7.11	5.5
*Sapium* sp.	1	3.07	3.87	0.27	0.4	8.92	9.74	2.76	1.22
*Schinopsis brasiliensis* Engl.	0.6	1.54	1.2	-	-	6.65	4.75	-	-
*Spondias tuberosa* Arruda	1	0.29	0.67	0.16	0.66	1.83	3.41	1.2	4.84
*Syagrus* sp.	1	2.11	2.14	-	-	39.98	6.61	-	-
*Handroanthus impetiginosus* Mattos	0.68	2.78	2.27	0.22	0.22	9.86	8.84	2.33	0.56
No identified plant	1	0.1	0.13	-	-	0.49	1.48	-	-

PA1 = Results for the sample plot method for Area 1; QA1 = Results of the point-centered quarter method for Area 1; PA2 = Results for the sample plot method for Area 2; QA2 = Results of the point-centered quarter method for Area 2, in *Sítio Carão*, Altinho municipality, Pernambuco (NE Brazil).

There was no correlation between RD, UV and IV for the sample plot method in Area 2 (rs = - .37, p = 0.14 and rs = -0.2, p = 0.41, respectively). However, there was negative correlation between RD, UV and IV for the point-centered quarter method in Area 2 (rs = - .53, p = 0.02 and rs = -0.59, p = 0.0008, respectively). Consequently, species with the highest UV had the lowest RD and IV (Table 2). Among them, *Libidibia ferrea* (Mart. ex Tul.) L.P. Queiroz (Pau-ferro), *Chorisia speciosa* A.St.-Hil. (Barriguda) and *Erythrina velutina* Willd. (Mulungu) showed the highest UV (above 1) and the lowest RD (0.13%). In relation to IV, these species correspond to only 3.34% of the total IV. It is evident that there are significant relationships between the UV and phytosociological parameters, depending on the location and the type of vegetation sampling.

## Discussion

### Methods used in ethnobotanical studies for accessing local knowledge about native plants

The participatory workshop resulted in a greater number of errors in the identification of plants, which is related to the biological material being presented outside of the original plant environment. By using exsiccates and photographs as visual stimuli, plant naming and identification become more problematic because the botanical and ecological details are absent [16,35]. Photographs in particular are difficult because the informant does not have access to the tactile and olfactory information necessary for the accurate identification of plants [18]. The recognition of plants through pictures can be improved if an object of known size or dimension is placed beside the biological material before photographing. Another feature that can be useful in the recognition of species is making a cut on the plant’s bark, a procedure used by many people in the field to aid in the recognition of plants.

Therefore, in situations in which one wishes to know the identity of a plant, the recommendation is to pay attention to the photographs and exsiccates used as visual stimuli. However, if the plant identity has already been established, these tools can be effective for obtaining more information about the plant, especially in cases in which the community member is elderly or would have some difficulty in being relocated to the field [16].

Unlike the use of photographs and exsiccates in participatory workshops, the inventory interview has the same advantage as the guided tour reported by Medeiros et al. [40]: they both provide for the observation of the plant in its unique biological and ecological context. The inventory interview included more citations of species recorded in the vegetation because ecological characteristics are of great importance in the recognition of plant species by parataxonomists [8,14]. The only limitation would be the difficulty of relocating elderly informants or those with limited mobility to the field.

Consequently, the involvement of parataxonomists is of great importance in the diagnostic process of local flora. Janzen and Hallwachs [41] argue that despite their lack of formal higher education, parataxonomists have shown themselves to be a group of great interest because they are able to absorb and work around the complex factors related to biodiversity, providing accurate inventory at levels similar or superior to those provided by undergraduate and postgraduate students. Jinxiu et al. [8] compared the recognition of plant species by taxonomists and parataxonomists on a given field over a year and found that the parataxonomists were able to recognize more plant species than the taxonomists. Cunha and Albuquerque [42] found that local informants recognized more than 95% of the plant species presented to them.

Among the limitations of involving parataxonomists would be the fact that they recognize only those species within their cultural field or those that they have encountered through their personal experiences [14]. Thus, the involvement of parataxonomists in rapid assessments is recommended, especially on occasions in which there is a pre-existing inventory of species found in the field.

The identification of the plants recognized by informants is vital to avoiding the distortion of future references. This would be an advantage of presenting the biological material in its original context, as the researcher is able to ensure the identity of the plant cited. It is important for the researchers to pay close attention when conducting in-home interviews, especially when no pre-interview research has been performed because one botanical species can be associated with several names and because several species may have the same popular name [9-11].

By comparing the plants recorded at *Sitio Carão* with those from other regions, it has been found, for example, that *Rhamnidium molle* Reissek is known as *sassafras*; in contrast, the Fulniô Indians in the Águas Belas municipality in Pernambuco recognize the species *Ditaxis malpighiacea* (Ule) Pax e K. Hoffm as *sassafras *[22]. *Allophylus quercifolius* (Mart.) Rasdlk. is recognized as *estralador* in the community of *Riachão de Malhada de Pedra*, Caruaru municipality in Pernambuco [43], though *estralador* is a species of Myrtaceae for the residents of *Sitio Carão*. Thus, the validity or consistency of popular names will be limited to each location.

In this study, the high richness of species exclusively recorded in semi-structured interviews cannot be interpreted as an advantage of this technique because the other methods used in this comparison (inventory interview and participatory workshop) were dependent on the diversity recorded in vegetation sampling. Thus, the sample size of the vegetation inventoried in this study (0.8 ha) may have been insufficient to register a greater richness of native species. By consequence, the informants who participated in the participatory workshop and inventory interview may have been introduced to a lower species richness that is not representative of what they actually know. Added to this, the time effort devoted to semi-structured interviews was higher than in other methods.

Although the inventory interview requires more time for execution, it is the most recommended method because it not only enables a more complete record of the species occurring in the region but also ensures more accurate identifications. For researchers to reduce the amount of time it takes to conduct a vegetation inventory, they may choose to use the point-centered quarter method because it requires less time in its application and fewer workers in the field [44,45]. Another useful recommendation for selecting the locations of units to be used for vegetation inventory is the deployment and distribution of the units in landscapes with different histories of disturbance to accommodate the largest possible number of environments and plant species [46-48].

### Relationship between use value and vegetation structure from the application of different methods for inventorying the vegetation in places with different histories of disturbance

Many of the species identified using these methods are among the least abundant or have a smaller IV and an elevated UV. This is due to the cultural prominence of the species or to the recognition of these species in the active memories of the participants, and they are therefore more frequently cited or remembered. This prominence can be related to the well-known presence of these plants, which is not necessarily related to their abundance, their stature or size [14], their inner properties (e.g., medicinal properties due to chemical composition), or cultural preferences, such as fashion or tastes. Thus, species with low abundance or IV may have a higher UV due to being cited more often. For example, *L. ferrea* (Pau-ferro), *C. speciosa* (Barriguda) and *E. velutina* (Mulungu) have low values of RD and IV (Table 2); however because these are large species, they are better known in the community, which facilitates their recognition, recall and utility and consequently raises their UV.

The relationship between the vegetation structure and the use value of plant species still lacks a clear-cut pattern in the scientific literature. Although the purpose of this study was to show the influence of methodological choice in studies about ecological apparency for more clarification about the ecological apparency hypothesis see [48], it was realized that this relationship was not significant in the community of Sítio Carão and that insignificant relationship is independent of the ecological tool used or the level of disturbance of the sample surveyed area. However, some studies have identified significant and positive relationships between use and availability of plant resources [49-51]. A recent example can be seen in Gueze et al. [52], where in a random vegetation sample by plots the authors found significant relationships between the use value and the ecological importance of tree species in the Bolivian Amazon, providing further support to the hypothesis that people attach more uses to the species that are more apparent in the forest.

The use of semi-structured interviews allows the recognition of the most prominent species of a particular cultural field [53,54], and these species are cited because they are present in a person’s active memory. Incorporating the semi-structured interview as a tool for the rapid assessment of diversity is an excellent method for species inventories if there is no pre-existing inventory of vegetation, which also allows UV calculation. The use of a pre-existing vegetation inventory may allow for easier recognition of the species, as it would provide availability to previously recognized vegetation.

For some researchers, the structural importance of certain taxa was correlated with their UV [13], whereas other groups have concluded that not all species are used in accordance with high values of phytosociological parameters [24]. According to the results observed in this study, these differences may be related to the location and methods chosen for sampling vegetation. Galeano [13] used transects extended over 10 km, and Tacher et al. [24] used secondary data of species recorded in Mexico. To select areas with the largest number of species, the researcher should select sites that are well preserved. The point-centered quarter method is a sampling tool that is useful in this regard, in addition to being faster than the sample plot method [43,44].

## Conclusion

Among the ethnobotanical methods assessed in this work, the inventory interview proved to be the best for registering local species. Although it requires more time and investment, as it is necessary to perform a vegetation inventory, there can be a greater guarantee of correctly identifying the botanic species; furthermore, the informant is able to better identify the plants presented, even if they were not identified during participative workshops.

If researchers wish to collect information from the elderly or other members of the community who may have difficulties performing the interview in situ, they may present visual stimuli, such as photographs or exsiccates. However, researchers should offer additional information to the informants to facilitate their recreation of the plant’s original environment.

In cases in which immediate ethnobotanical inventory is necessary, researchers can opt to hold a census by means of semi-structured interviews conducted with all the adults of the community or with a representative group of this population. However, it needs to be accepted that only the most prominent species and those most easily remembered will be cited, while other species found in the community will most likely not be included in the inventory. For researchers to optimize their time, it is better to utilize the point-centered quarter method than the sample plot method. It is also recommended that samples from different areas in the landscape with different histories of disturbance be gathered to record as many different environments and species as possible.

## Competing interests

The authors declare that they have no competing interest.

## Authors’ contributions

All authors participated in the design of the study and the writing of the paper. All authors read and approved the final manuscript.
